# Cooperative standing-horizontal-standing reentrant transition for numerous solid particles under external vibration

**DOI:** 10.1038/s41598-017-18728-6

**Published:** 2018-01-11

**Authors:** Satoshi Takatori, Hikari Baba, Takatoshi Ichino, Chwen-Yang Shew, Kenichi Yoshikawa

**Affiliations:** 1 Faculty of Life and Medical Sciences, Doshisha University, Kyotanabe, Kyoto 610-0394 Japan; 2 Faculty of Biology-Oriented Science and Technology, Kindai University, Kinokawa, Wakayama 649-6493 Japan; 3 Ph.D. Program in Chemistry, The Graduate Center of the City University of New York, New York, NY 10016 USA; 4 Department of Chemistry, College of Staten Island, Staten Island, NY 10314 USA

## Abstract

We report the collective behavior of numerous plastic bolt-like particles exhibiting one of two distinct states, either standing stationary or horizontal accompanied by tumbling motion, when placed on a horizontal plate undergoing sinusoidal vertical vibration. Experimentally, we prepared an initial state in which all of the particles were standing except for a single particle that was placed at the center of the plate. Under continuous vertical vibration, the initially horizontal particle triggers neighboring particles to fall over into a horizontal state through tumbling-induced collision, and this effect gradually spreads to all of the particles, i.e., the number of horizontal particles is increased. Interestingly, within a certain range of vibration intensity, almost all of the horizontal particles revert back to standing in association with the formation of apparent 2D hexagonal dense-packing. Thus, phase segregation between high and low densities, or crystalline and disperse domains, of standing particles is generated as a result of the reentrant transition. The essential features of such cooperative dynamics through the reentrant transition are elucidated with a simple kinetic model. We also demonstrate that an excitable wave with the reentrant transition is observed when particles are situated in a quasi-one-dimensional confinement on a vibrating plate.

## Introduction

There is a growing interest in spatiotemporal self-organization under thermodynamically open conditions in a wide range of disciplines in the natural sciences. Among many experimental systems on spatiotemporal dynamics with far-from-equilibrium conditions, the behavior of bouncing objects generated by mechanical agitation is particularly interesting because of its simplicity and the ease at which control parameters can be changed^1–10^. Many novel dynamical behaviors have been identified experimentally for single non-spherical particles. Khan *et al*. showed that symmetric vibration induces the locomotion of wet granular particles of an asymmetric dimer consisting of two spheres of different sizes in a tube that is aligned vertical with respect to gravity^1^. Yamada *et al*. reported that a single asymmetric bolt particle confined between two plates exhibits random motion or unidirectional motion depending on the strength and frequency of vertical vibration, and this behavior is insensitive to the detailed morphology of the particle^2^. Dorbolo *et al*. applied vertical vibration to a single dumbbell and observed horizontal drifting, or vectorial motion, where one end stays on the vibrating plate most of the time while the other end bounces^3^. Kubo *et al*. introduced chiral asymmetry to a dumbbell and found characteristic mode bifurcation for the distinct motions of the asymmetric dumbbell, including random fluctuation, orbital motion and rolling^4^. Wright *et al*. investigated the behavior a single rod under vibration^9^, and found the rod underwent a transition from periodic motion to stochastic motion by increasing the acceleration of the vibration^9^. In relation to the unique characteristics of rod granular particles, Trittel *et al*. reported the marked differences between the bouncing statistics of spherical particles and cylinders^10^.

In addition to studies of single particles under vibration, vibration experiments with multiple particles have been carried out to gain insight into their collective behavior^9,11–14^, together with a study on a dense suspension of particles^15^. Several studies have reported the dynamic behavior of numerous particles in terms of crystallization^16–19^, the glass transition^20,21^, and a first-order phase transition with phase-coexistence^22^, by noting similar characteristics under thermal equilibrium. Some reports^23–25^ have described marked differences from the characteristics under equilibrium thermodynamics. Müller *et al*. showed that a monolayer of wet granular rods under vibration form uniaxial nematic and tetratic phases depending on their aspect ratios^26^. For the monolayer of wet spherical granular particles, May *et al*. used two combined horizontal vibrators, and found that vibration drives the surface to be melted away from equilibrium, and the system needs to pass an amorphous state prior to the transformation of crystal into liquid state^27^. While a monolayer of wet spherical granular particles is under vertical vibration, Zippelius *et al*. observed wave-like fronts due to the instability of the gas-liquid-like transition, circling around the rim of the cylindrical cavity^28^. In fact, propagating waves were also identified by using the monolayer of fluidized granular rods through air up-flow^29^. It is also noted that vibration experiments in microgravity have been applied to granular rods and spheres. In dilute rods, their translational and rotational energies were non-Gaussian distribution indicating non-equilibrium nature^30^. In 3D cooling, the equipartition between translational and rotational energies of granular rods was violated^31^. For granular spheres, Sack *et al*. reported synchronized motion between granular particles and the driven wall at high vibration amplitudes, but at low vibration amplitudes, granular particles exhibited gas like behavior^32^. When the granular particle density become high, Falcon *et al*. noticed the motionless cluster formation which is surrounded by the region of low particle densities^33^. Deseigne *et al*. investigated a monolayer of vibrating disks with polar asymmetry. As the amplitude of vibration increased, the asymmetric polar particles underwent a change from alignment to large-scale fluctuation^5^. Oda *et al*. recently used vertical vibration to study the 2D structure of a layer of small spherical granular particles mixed with larger particles confined within a cylindrical cavity^34^. They demonstrated that, with an increase in the degree of crowding of small spheres, the preferred position of the large particles changes from the region near the cavity wall to the interior of the cavity. In addition, wave-like propagation has been shown to be generated for granular particles under external agitation^35,36^. Granular particles driven by mechanical vibration are also considered to be a representative real-word model of so-called active particles^37^.

In this work, we investigated the collective dynamical behavior of asymmetric plastic bolt-like particles confined within a cylindrical cavity under vertical vibration. Bolt-like particles display two distinct states: standing stationary and horizontal with tumbling motion. We observed the temporal evolution of the transition between the two states. We found that vertical vibration first triggers the transitions of neighboring particles from standing to horizontal and then from horizontal to standing, as a result of collisions with tumbling horizontal particles. Interestingly, under an appropriate vibrational intensity, we found that this reentrant behavior causes phase segregation between standing particles that show symmetric packing and those under disordered dispersion. In addition, an excitable wave accompanied by the standing-horizontal-standing reentrant transition is observed under quasi-one dimensional constraint for the assembly of the particles.

## Results and Discussion

### Behavior of a single bolt-like particle

**Figure 1:**
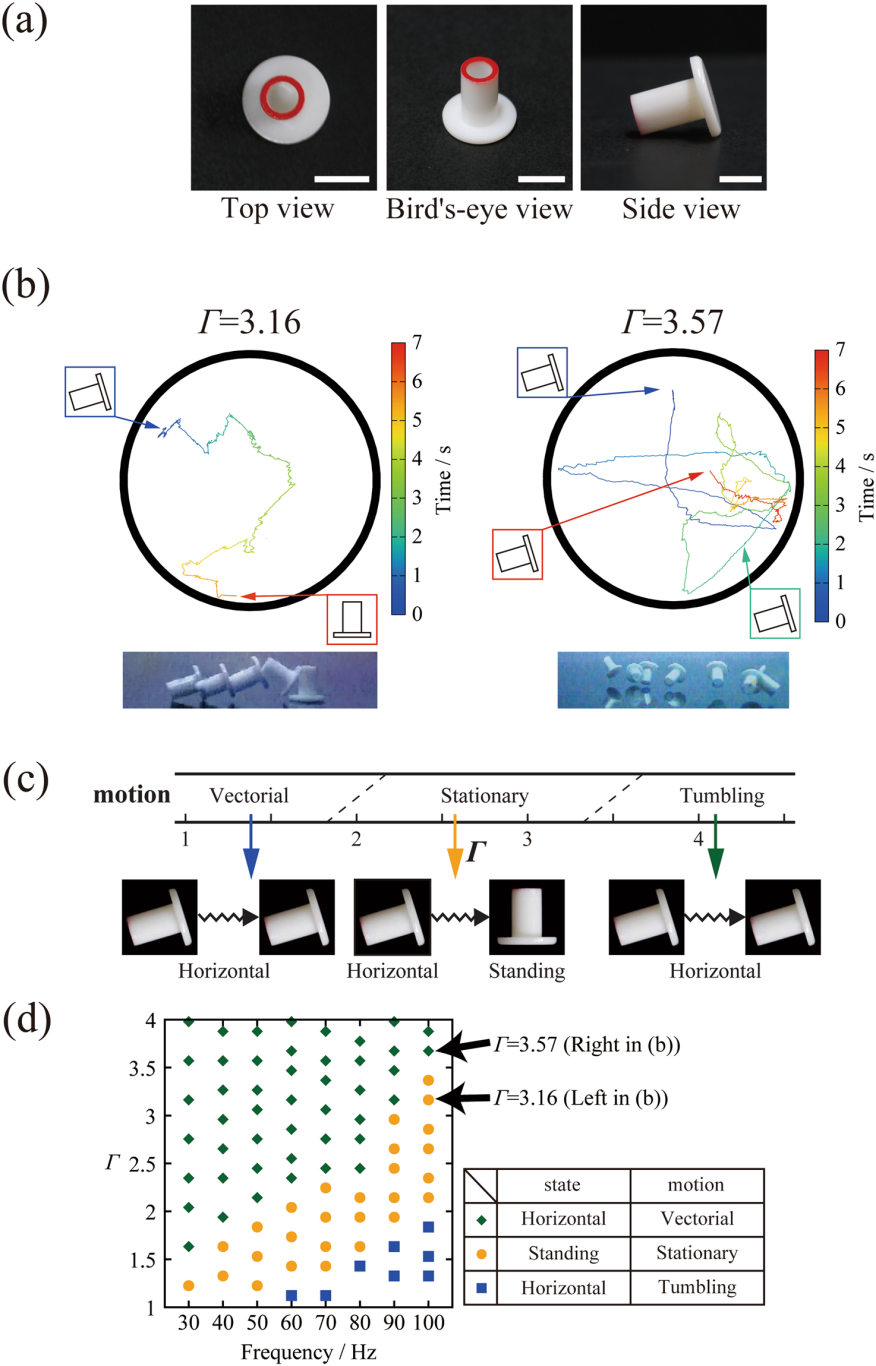
Behavior of a single bolt-like particle driven by vertical vibration. (**a**) Photos of the bolt-like particle used in the present study; bar is 5 mm. (**b**) Examples of characteristic time traces of a single bolt-like particle at different *Γ* values. The vibration frequency of the plate was fixed at 100 Hz. The horizontal particle stands up spontaneously during tumbling motion at *Γ* = 3.16, whereas at *Γ* = 3.57 the horizontal state with tumbling motion continues during vertical agitation. It is noted that “Standing” and “Horizontal” correspond to the actual observation shown in the left and right of (**b**), respectively. The bottom picture are the overlaps of snapshots in the actual observations. (**c**) The state of the motion of a single bolt-like particle as a function of *Γ* at a fixed frequency, *f* = 100 Hz. (**d**) The Phase diagram with *Γ* vs. frequency for the state of motion of a single bolt-like particle.

Figure 1a shows a schematic view of the experimental system (left) and photos of a bolt-like particle used in the present study. To indicate the strength of the forced vibration, we use dimensionless acceleration amplitude, ${\Gamma }={A}{(2{\rm{\pi }}f)}^{2}/{g}$, where *A* is the amplitude of oscillation and *g* is gravitational acceleration. Prior to the experiments involving a large number of particles, we examined the behavior of a single bolt-like particle under different amplitudes, i.e., different *Γ* values (for details, see the Supplementary data). Figure 1b depicts the characteristic modes of the spontaneous motion of a single bolt-like particle under two distinct conditions: *Γ* = 3.16 (*A* = 0.078 mm, *f* = 100 Hz) and 3.57 (*A* = 0.091 mm, *f* = 100 Hz). We confirmed that, with an increase in *Γ*, the particle exhibits a transition from vectorial motion in a horizontal state to a situational standing state, and finally to a horizontal state with random tumbling motion. Figure 1c shows a diagram of the mode of a single particle depending on the strength of vibration, *Γ*; vectorial motion in the horizontal state is seen when *Γ* < ca.2.0, standing up spontaneously from the horizontal state to the stationary standing state is seen when ca. 2.0 < *Γ* < ca. 3.7, and the horizontal state with random tumbling motion is seen when *Γ* > ca. 3.7. Figure 1d plots the phase diagram in terms of *Γ* vs. frequency for the state of motion of a single bolt-like object. With an increase in *Γ*, mode bifurcations develop: vectorial motion in the horizontal state, standing state, and horizontal state with continuous tumbling motion. Notably, standing state is stable regardless the initial condition, either standing or horizontal. Based on these results shown in Fig. 1a–d, we have extended our study to the collective motion of multiple bolt-like particles by choosing the condition at *f* = 100 Hz, in which condition the mode-bifurcations are caused in an apparent manner.

### Cooperative motion of numerous particles

**Figure 2:**
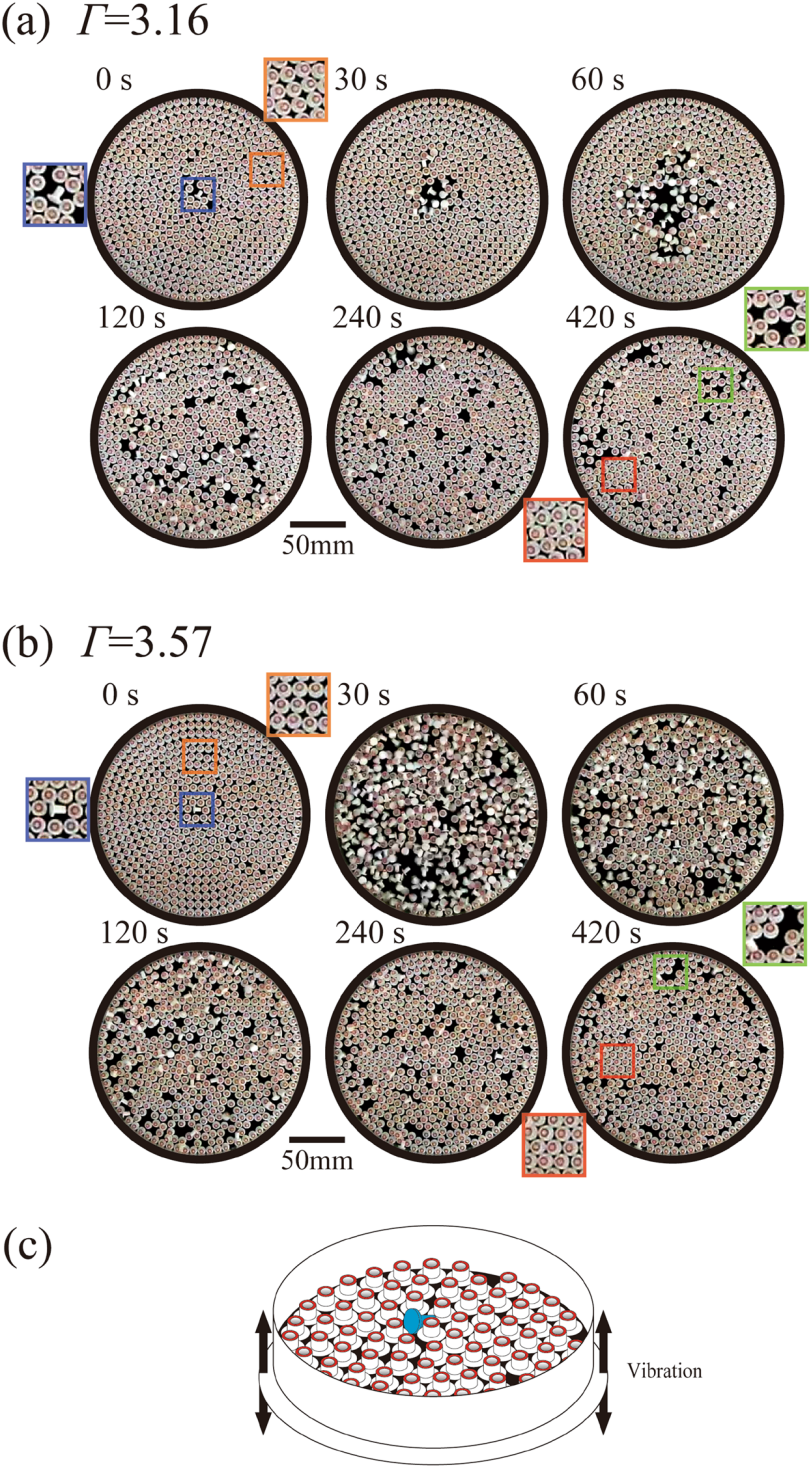
Time-successive snapshots of the behavior of 521 particles, indicating a reentrant transition. In the initial condition, t = 0, a single particle was placed horizontaly. The frequency was fixed at 100 Hz (**a**) *Γ* = 3.16(Amplitude, 0.078 mm), (**b**) *Γ* = 3.57(Amplitude, 0.091 mm). In both experiments, the particles initially tend to fall over into the horizontal state sate and undergo tumbling motion. Next, horizontal particles gradually change to the standing state through collisions with neighboring particles. (**c**) Schematic representation of the bolt-like particles (n = 521) arranged in a circular vessel with a diameter of 197 mm, which is placed on an aluminum plate. Sinusoidal vibration was applied in the up-down direction.

Figure 2 shows an example of the collective motion of 521 bolt-like particles on the vibrating plate confined within a cylindrical cavity (197 mm in diameter) for *Γ* = 3.16 and 3.57, respectively (the Supplementary materials include two video files; Video 316.avi and Video 357.avi). At time t = 0 s, Fig. 2a and b both start with 520 bolt-like particles in a standing state near-uniformly distributed around the vibrating plate, after having been arranged manually, and one particle is placed horizontally at the center of the plate. In Fig. 2a, the number of horizontal particles undergoing tumbling motion gradually increases with time, due to the collision through the random tumbling motion of the horizontal particles with neighboring standing particles, as exemplified at t = 30 and 60 s. At t = 120 s, the horizontal particles near the central region begin to reorganize into a standing state, i.e., a reentrant transition of standing-horizontal-standing. As time proceeds to 240 and 420 s, the system exhibits the features of a steady state where the majority of particles are in the standing state, while a small number of particles remain in the horizontal state. When the vibration experiment is performed at a higher *Γ* (= 3.57), as shown in Fig. 2b, similar but more extreme time-dependent changes in the particle states are found. This difference is attributable to the fact that a greater *Γ* accelerates the transformation of the standing particles to the horizontal state. As a result, at t = 30 s, almost all of the particles are in the horizontal state. Between t = 30 and 60 s, the horizontal particles quickly reorganize to the standing state. From t = 120 to 420 s, the system reaches the steady state, and only a small number of particles remain in the horizontal state. The results of these experiments are insensitive to the exact position of individual particles in the initial configuration when the number of particles is set constant (data not shown).

In these experimental runs for both high and low *Γ* values, we set the initial condition so that 520 particles were standing and a single particle near the center of the plate was placed horizontally, i.e., the total number of particles was 521. We placed the particles manually to create a circular arrangement from the outer region toward the interior, as depicted in the figures at t = 0 s. The area occupied by all 521 particles under the standing state was ca. 85.9% of the entire area of circular confinement. Since the area occupied by densely packed hexagons is 90.7%, the number of standing particles that exhibit dense-packing in a perfect hexagonal lattice is calculated to be 521 · (90.7/85.9) = 550 in the area of our vibrating plate. Thus, in the experiments with 521 particles, the surface has some vacant space that can accommodate an additional 29 standing particles.

A careful inspection of Fig. 2 suggests that, in the steady state after the reentrant transition, the standing particles exhibit a tightly packed 2D hexagonal structure. Thus, one may argue that the formation of a cluster with standing particles that exhibit a more compact 2D hexagonal structure in the steady state is essential for stabilizing the ordered standing phase.

### Change in symmetry between before and after the reentrant transition

**Figure 3:**
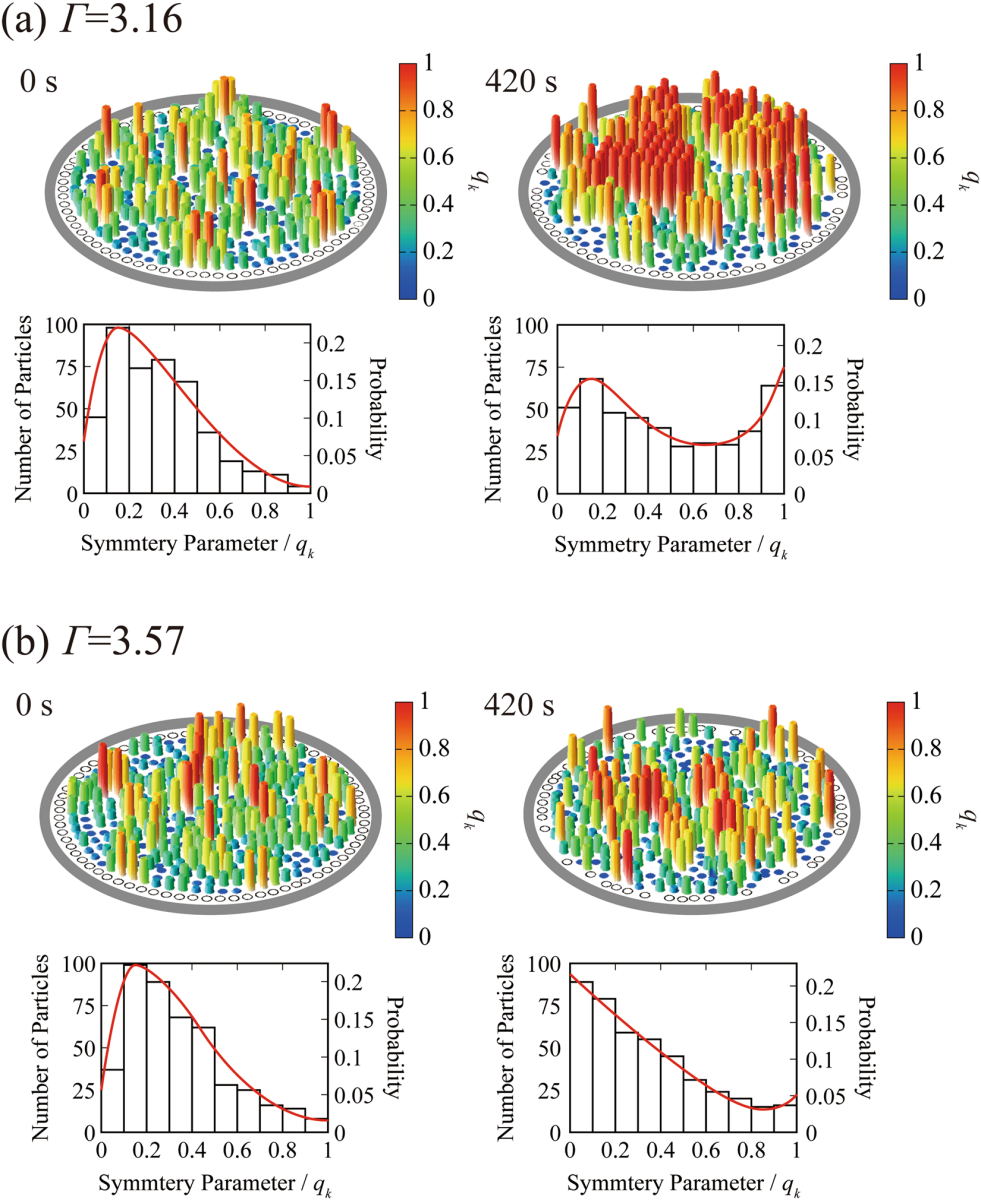
Change in the symmetry parameter before and after the reentrant transition. (**a**) Spatial distribution of the symmetry parameter *q*_*k*_ for standing particles in the initial state and the almost-stationary state after the reentrant standing-horizontal-standing transition for the experiments shown in Fig. 2. The pseudo color indicates the symmetry parameter deduced from equation (1). Below each color map, the probability of the symmetry parameter is shown in the histogram together with a red fitting curve. (**a**) *Γ* = 3.16 (**b**) *Γ* = 3.57. At *Γ* = 3.16, the unimodal distribution at the initial condition changes to a bimodal profile at t = 420 s, after the reentrant transitions of the particles, which corresponds to the fundamental characteristics of a first-order phase transition.

To better understand the mechanism of the reentrant transition, we evaluated the nature of the spatial packing of particles by comparing the initial state (t = 0 s) and the steady state after the reentrant transition (t = 420 s). Figure 3 shows the results of our analysis of the degree of hexagonal symmetry. We evaluated the spatial correlation among six nearest-neighboring particles by using the following relationships^38,39^:

1 $${q}_{6}^{(k)}:=\frac{1}{6}{{\rm{\Sigma }}}_{j\in {N}_{6}^{(k)}}{e}^{i6{\alpha }_{kj}}$$

2 $${q}_{k}={|{q}_{6}^{(k)}|}^{2}\in [0,1]$$

where *q*_*k*_ is the symmetry parameter that reflects the local six-fold orientational order of particle *k*. In equation (1), the sum is taken over the six nearest neighbor’s *j*’s for particle *k*, and *α*_*kj*_ is the angle between **r**_(k)_–**r**_(j)_ and a randomly chosen axis. For convenience, we show the particles adjacent to the wall of the circular confinement as white circles, because the boundary wall disrupts the continuation of hexagonal symmetry. In Fig. 3a for *Γ* = 3.16, the initial configuration at t = 0 s shows some weak hexagonal packing (loose packing) on the vibrating plate in a random fashion, denoted by yellow and blue. At t = 420 s the pattern of close hexagonal packing emerges as “islands” denoted by red and yellow, connected by a loosely packed hexagonal structure, denoted by yellow and blue. For a higher value of *Γ* ( = 3.57), Fig. 3b shows smaller hexagonal domains of hexagonal structure compared to those in Fig. 3a. For the higher *Γ*, the force of collision is greater during reentrance to the standing phase, which may interfere with the formation of a large domain of hexagonal packing. In Fig. 3, the measured values of the symmetry parameter, *q*_*k*_, are also given for t = 0 s and 420 s. It is clear that the number of particles with higher symmetry or dense packing increases after the reentrant transition and that more particles form genuine hexagonal packing under the smaller *Γ*. For *Γ* = 3.16, as in the histogram of Fig. 3a, the distribution concerning the degree of six-fold symmetry indicates the transition from a unimodal profile at t = 0 s to a bimodal profile at t = 420 s. A similar but somewhat unclear trend is also encountered for *Γ* = 3.57 in the histogram of Fig. 3b. These results suggest that the final stationary states exhibit the characteristics something similar to the phase-segregation generated under first-order phase transition.

**Figure 4:**
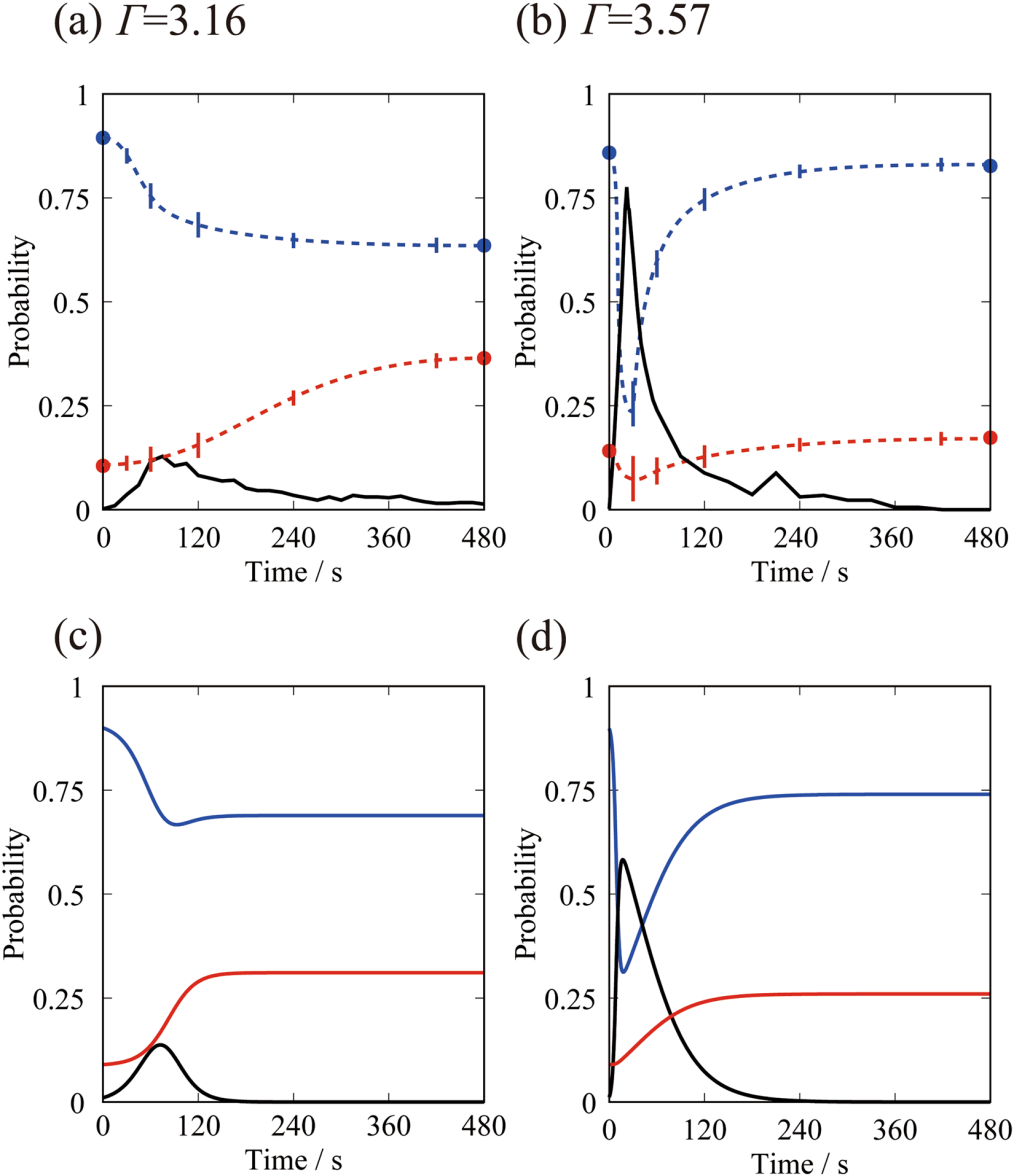
Experiments (**a**,**b**) and numerical simulations (**c**,**d**) on the time-development of the states of the particles. (**a**,**b**) The solid line represents the probability of tumbling particles. The broken blue and red lines are guides for the eye to show the changes in the number of standing particles below and above a value of 0.6 for the symmetry parameter *q*_*k*_, which correspond to disordered and ordered states, respectively. (**c**,**d**) Numerical results for the time evolution of the three different states: standing with low symmetry, horizontal with tumbling motion, and standing stationary with six-fold rotational symmetry.

Figure 4a and b show the time-dependent change in the number of horizontal particles, denoted by solid lines, for agitation with *Γ* = 3.16 and 3.57, respectively. As a guide for the eye, we show dashed lines to indicate the time progress of standing particles with lower (*q*_*k*_ < 0.6), denoted by blue line, and higher symmetries (*q*_*k*_ > 0.6), denoted by red line. For both of the *Γ* values, the experimental traces indicate that state of the particles attains a stationary phase for the time after around t = 400 s. Both figures also show a primary peak followed by a gradual decay along with some perceivable fluctuation in the number of particles in the horizontal state. Figure 4a shows a weaker but slightly wider peak (measured from the half-height width) with a maximum at around t = 70 s, which corresponds to about 15% of particles in the horizontal state. These horizontal particles tumble more locally around the central area of the circular plate, as shown in Fig. 2. In contrast, Fig. 4b shows a stronger but slightly narrower peak at about t = 20 s, where close to 80% of particles are in the horizontal state, evenly fluctuating across the entire vibrating plate. The small number of horizontal particles continues to decay appreciably at around t = 300 s, and before t = 420 s, the system has only a few horizontal particles. This result indicates that a greater *Γ* accelerates the tumbling process more strongly, and more particles enter the horizontal state at the early stage. Next, we consider the possible mechanism that stabilizes the standing particles accompanied by an increase in the densely packed state.

Reentrant transitions are found in many other systems, such as non-Boussinesq convection^40^ and oscillatory chemical reactions^41^. For non-Boussinesq convection, a temperature gradient induces the formation of hexagons due to the instability of the fluid at the onset of convection. As the system approaches the critical point, reentrant hexagons appear only within a narrow regime^40^. In our system, the reentrant standing phase also occurs within a certain window for *Γ* around 3.2 to 3.6, and the initial standing phase is unstable upon vertical vibration. Beyond this range, the system stays in the loosely packed standing phase for smaller *Γ*, or all particles fall to the horizontal state for greater *Γ*. Interestingly, in our experiments, fluctuation caused the reentrant transition by inducing regular hexagonal packing.

### Numerical Modeling

To better understand our experimental observation, we established a simple kinetics model consisting of three states: (i) *x*: standing in a dispersed state without 2D hexagonal close-packing; (ii) *y*: horizontal state; (iii) *z*: standing state stabilized through dense-packing. Based on a consideration of the time-dependent change in the state of particles through the reentrant transition, we propose the following simple kinetic model:

3 $$\frac{dx}{dt}=-{k}_{1}xy+{k}_{3}yz$$

4 $$\frac{dy}{dt}={k}_{1}xy-{k}_{2}yz$$

5 $$\frac{dz}{dt}={k}_{2}yz-{k}_{3}yz$$

where *k*_1_, *k*_2_ and *k*_3_ are the rate constants for three different kinetic pathways that regulate the fractions of the three states over time. For simplicity, we adopt the approximation that all of the kinetic processes can be interpreted as being the result of collision, i.e., the kinetics are given as the product of the variables *x*, *y*, and *z* under the condition that *x* + *y* + *z* = 1. The fraction *x* is decreased through collision with a horizontal particle in state *y*, but is increased when particles in state *y* enter state *z*, and *z* changes to *x*. The transition of a standing particle to the horizontal state is triggered by collision with a randomly tumbling horizontal particle, as represented in the term *k*_1_ *xy*. When the collision energy is high enough, standing particles fall into the horizontal state. We hypothesize that a horizontal particle, *y*, flips to the standing state by colliding with the cluster of standing particles under hexagonal close-packing, *z*, i.e., the term *k*_2_ *yz*. We also consider the kinetics of the production of a loosely packed or isolated standing state through the collision between a horizontal particle, *y*, and a standing particle with tight packing, *z*, as in *k*_3_ *yz*, where the horizontal particle changes into the standing particle in a dispersed state without the transition of the standing particles under tight packing. In other words, based on the careful observations for the experiments, we have noted that the collision between a horizontal plate, *y*, and cluster of standing particles under dense packing, *z*, causes two different processes; i.e., if) a horizontal particle changes into a standing state by joining onto the cluster of standing particles under dense packing, and ii) a standing particle belonging to a cluster of dense packing eliminates and changes into a standing state in a dispersed state. The former and latter processes correspond to *k*_2_ *yz* and *k*_3_ *yz*, respectively.

Note that other kinetics steps are neglected, such as collisions between particles and the confining wall, which are less important than the above mechanism under our experimental conditions. We also adopt the assumption that the kinetics do not involve collisions of more than two bodies. With this simple model with minimum variables, we calculate the time-dependent change in the probabilities of the three different states.

For the analysis of the symmetry parameter *w*_*k*._ in Fig. 3, we evaluate the parameters by eliminating particles adjacent to the wall. Thus, the probabilities of the different states in Fig. 4a,b were calculated for 466 particles. We tentatively classified standing particles with lower (*q*_*i*_ < 0.6) and higher symmetries (*w*_*k*._ > 0.6), corresponding to the probabilities of *x* and *z*. Accordingly, we used *x*(t = 0) = 0.9, *y*(t = 0) = 0.01, and *z*(t = 0) = 0.09 as the initial condition for the numerical simulation. In Fig. 4c, we show the numerical results corresponding to the experiments with *Γ* = 3.16, where *k*_1_ = 7, *k*_2_ = 29 and *k*_3_ = 20. On the right, the time evolution corresponds to the experiment with *Γ* = 3.57, where *k*_1_ = 50, *k*_2_ = 150 and *k*_3_ = 148. In order to find the fitting parameters, initially we have searched the suitable parameters from the initial stage for *k*_1_ and final stage approaching stationary phase for *k*_3_, respectively. Then, by changing the parameter *k*_2_, we tried to find the best parameters so as to reproduce the experimental trends. Figure 4 shows the probabilities of *x*, *y*, and *z* as a function of time for *Γ* = 3.16 (Fig. 4a) and 3.57 (Fig. 4b). Our calculations in Fig. 4c,d show that the model qualitatively agrees with the experiment as shown in Fig. 4a,b. For a greater *Γ*, a shorter time is sufficient to reach the peak of *y*, which also has a greater peak height, indicating that horizontal particles emerge more quickly. The model further predicts how *x* and *y* vary with time. With an increase in *Γ*, the system arrives at the steady state more rapidly and the number of standing particles in contact with closely packed standing particles increases. From the model study, we find that the rate constant *k*_2_ is critical for determining the speed of the reentrance of bifurcation to the stable standing phase. Moreover, *k*_2_ has a direct impact on the steady state. A smaller *k*_2_, corresponding to a smaller *Γ*, causes less fluctuation in the system. Consequently, more loosely packed standing particles remain in the system, which is related to the more frequent transformation coupled with greater numbers of coexistent standing and horizontal particles. In contrast, a greater *k*_2_, corresponding to a greater *Γ*, is associated with a greater degree of fluctuation around the entire vibrating plate. This leads to a smaller fraction of particles in a tightly packed hexagonal arrangement among the standing particles after the reentrant transition.

### Generation of a traveling wave in a quasi-one-dimensional system

**Figure 5:**
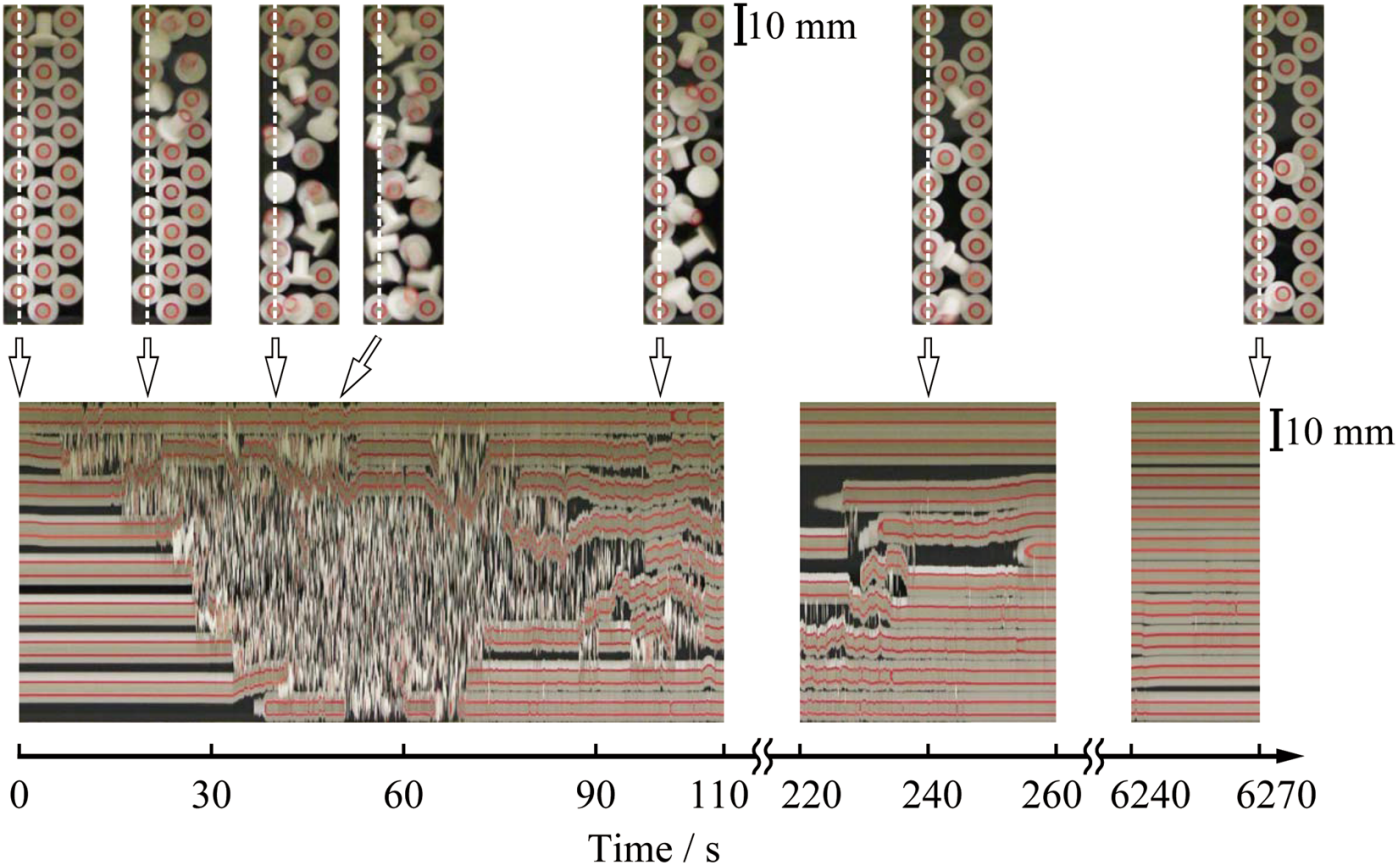
Appearance of a traveling wave in a quasi-one-dimensional system. The upper pictures are the actual arrangements of the particles that change with time under external vibration, and the bottom picture is a spatio-temporal diagram. The vibration frequency is 100 Hz and *Γ* = 2.75.

From the above discussion, it is clear that the reentrant transition of standing-horizontal-standing exhibits characteristics somewhat similar to those for the signal excitation of nerve impulses. Thus, as the next target, by considering the analogy to the propagation of excitation along a nerve axon, we examined the possible generation of an excitable wave or traveling wave by performing an experiment in a quasi-one-dimensional system as in Fig. 5, where we set, *Γ* = 2.75 (*A* = 0.078 mm, *f* = 100 Hz; see the video file onedimension.avi in the supplemental materials). When we start the vibration experiment by putting a single particle in a horizontal state, as in the top-left picture in Fig. 5, the propagation of horizontal particles is observed between t = 10 and 30 s. After the transition from standing to horizontal state, the reentrant transition also appears and propagates, although the reentrant process is less prominent. Finally, after 250 s, a regular standing pattern is generated and continues in a stationary manner. Thus, as a preliminary experiment, we observed the generation of a travelling wave for a quasi-one-dimensional system.

Traveling waves are generally induced for spatially distributed systems in which local elements exhibit excitability. As described in the preceding sections, under suitable conditions the plastic bolt-like particles undergo a transition from a standing state to a horizontal state, and eventually back to a standing state. The final standing state is characterized as dense packing with high spatial symmetry, whereas the initial state exhibits less spatial symmetry. Thus, we may consider such a transition a kind of excitable system. The essential feature of excitation can be described as a dynamical system with bimodality, or double-minima in the free energy profile. To describe excitability, we can adopt the following nonlinear kinetic equation^42^:

6 $$\frac{\partial u}{\partial t}=-\frac{\delta F}{\delta u} \sim -\sigma u(u-a)(u-1)$$

where $\frac{1}{2} < a < 1$. We can consider time development from *u* = 0 to *u* = 1 under excitation.

For one-dimensional propagation of an excitable wave, by considering the free energy functional with a spatial derivative of *u*, we can obtain

7 $$\frac{\partial u}{\partial {\rm{t}}}=D\frac{{\partial }^{2}u}{\partial {x}^{2}}-\sigma u(u-a)(u-1)$$

We may thus obtain a solution of a traveling wave with a constant velocity c^42^:

8 $${\rm{c}}={(\frac{{\rm{\sigma }}D}{2})}^{1/2}(2a-1)$$

By introducing the rate-determining rate constant *k*_0_, we can roughly rewrite equation (8) as^43^

9 $$c\, \sim \,\sqrt{{k}_{0}D}$$

Our inspection of Fig. 5 suggests that *k*_0_ is on the order of 0.02–0.03 s^−1^ for horizontal particles to stand up. From the time-dependent trace of a single tumbling particle, we may assume that the diffusion constant *D* is on the order of 1 cm^2^/s. Thus, from equation (9), the speed c should be on the order of several tenths of cm/s. The spatio-temporal plot indicates that c is on the order of 0.1–0.2 cm/s, which is consistent with the above expectation, although there exists relatively large fluctuation on the travelling wave.

The generation of a constant-speed traveling wave for solid particles has been observed in experiments on domino toppling^44,45^. However, as far as we are aware, there has been no report on the generation of an excitation traveling wave for a solid system. Although our observation of an excitable traveling wave is still in an early stage, we expect that the present study will inspire future studies to construct excitable traveling waves in more elegant experimental systems. This excitable wave may also attract interest in studies on informational operations with an excitable medium, as a novel type of non-Neumann and non-Turing computation^46,47^.

## Conclusions

We applied vertical vibration to many plastic bolt-like particles with two distinct orientation states (standing and horizontal) situated on a horizontal plate. In the initial condition, one particle near the center of the vibrating plate is placed in the horizontal state, and the other particles are in the standing state and exhibit loose packing. Upon vertical vibration, these loosely packed standing particles become unstable and enter the horizontal state mostly by colliding with tumbling horizontal particles. Based on our experiment and a model study, it has become clear that the strength of tumbling in the horizontal state determines the speed at which the system reenters the standing phase. The standing phase due to the reentrance of bifurcation is stabilized by the formation of crystalline domains, islands of 2D close-packing, that may open an energy dissipation pathway for the standing particles to help keep the crystalline domains with standing particles stable even under rather strong vibration. Thus, the observed formation of crystalline domains exhibits similar aspect as in spinodal decomposition through a first-order phase transition with the generation of dispersed regions surrounding mini-crystals. In an additional experiment (Fig. 5), we observed the generation of an excitation travelling wave with standing-horizontal-standing transition for the solid particles in a quasi-one-dimensional system.

In contrast to the recent studies regarding the phase transition of the monolayer of spherical granular particles in 1D and 2D vibration experiments with a clear interface between two phases^48,49^, the reentrance transition in our work is associated with a bifurcation process generated under far-from-equilibrium condition. At a high acceleration amplitude *Γ*, all of the bolt-like particles are under constant random tumbling. In a certain range of *Γ*, these particles exhibit reentrant transition and finally approach onto a quasi-stationary state, where nearly all particles undertake the standing state. The resulting quasi-stationary state is composed with solid-like dense packing and loosely packed regions. Being different from the phase segregation observed for usual first-order phase transition, there appears no clear interface in the resulting quasi-stationary state. One may argue that the time development of the observed system, i.e., attainment onto a quasi-stationary state through reentrant transition is a specific spatio-temporal structure generated under the condition of far-from-equilibrium.

## Methods

The plastic bolt-like particles consisted of a cylinder 6 mm in length and 4 mm in diameter attached to a circular plate of 1.5 mm in depth and 8 mm in diameter, and were purchased from Marusho Co. Ltd. (Osaka, Japan). Vibration was applied vertically to the aluminum bottom plate by an electromagnetic shaker (512 Series Vibration Generator; EMIC Co., Tokyo, Japan) with *z*(*t*) = *A*sin(2*πft*) at a fixed frequency, *f* = 100 Hz. The motion of particles was recorded with a high-speed camera (Model Ex-FH20, Casio, Japan).

## Electronic supplementary material

Collective motion for Γ = 3.16

Collective motion for Γ = 3.57

Travering wave for quasi-one-dimensional system

Supplemental information
