# Supplementary material for: Cooperative standing-horizontal-standing reentrant transition for numerous solid particles under external vibration
Source: Sci Rep. 2018 Jan 11;8:437. doi: 10.1038/s41598-017-18728-6 (PMC5765037; doi:10.1038/s41598-017-18728-6)
Supplement: Supplementary file 4 — Supplemental information [file 41598_2017_18728_MOESM4_ESM.pdf]

Cooperative standing-horizontal-standing reentrant transition for numerous solid particles under external vibration

Satoshi Takatori[1], Hikari Baba[1], Takatoshi Ichino[2], Chwen-Yang Shew[3] & Kenichi Yoshikawa[1]

[1] *Faculty of Life and Medical Sciences, Doshisha University, Kyotanabe, Kyoto 610-0394, Japan*

[2] *Faculty of Biology-Oriented Science and Technology, Kindai University, Kinokawa, Wakayama 649-6493, Japan*

[3] *Department of Chemistry, City University of New York, College of Staten Island, 2800 Victory Boulevard, Staten Island, NY 10314, USA*

Corresponding Author: Kenichi Yoshikawa

E-mail: keyoshik@mail.doshisha.ac.jp

Contents:

Supplementary videos

The Supplementary materials include three video files

Video S1.avi: This video shows an experimental observation on the collective motion at $\Gamma = 3.16$.

Video S2.avi: This video shows an experimental observation on the collective motion at $\Gamma = 3.57$.

Video S3.avi: This video shows an experimental observation on the generation of travelling wave accompanied by the reentrant transition of standing-horizontal-standing.
